# Pitfalls in interpreting red blood cell parameters in elite high‐altitude and sea‐level athletes: A unique case series

**DOI:** 10.14814/phy2.14891

**Published:** 2021-07-01

**Authors:** A. Mireille Baart, Jacqueline M. T. Klein Gunnewiek, Michiel G. J. Balvers, Johannes Zwerver, Peter C. J. Vergouwen

**Affiliations:** ^1^ Clinical Chemistry and Haematology Laboratory Gelderse Vallei Hospital Ede the Netherlands; ^2^ Department of Elite Sports Medicine and Sports Medicine Gelderse Vallei Hospital Ede the Netherlands; ^3^ Center for Human Movement Sciences University Medical Center Groningen Groningen the Netherlands; ^4^ Present address: Division of Human Nutrition and Health Wageningen University & Research Wageningen the Netherlands

**Keywords:** athletes, blood volume, hemoglobin, high altitude, red blood cells

## Abstract

Standard routine hematological measurements are commonly used to investigate differences in blood parameters between high‐altitude athletes (HAA) and sea‐level athletes (SLA), and to monitor the effect of high‐altitude training. In this way, red blood cell (RBC) parameters are usually expressed as *relative* parameters (concentration) rather than *absolute* parameters (total amount). In this unique case series of elite HAA and SLA, we describe how different ways of parameter expression can affect the interpretation of blood tests. In a group of 42 elite athletes, relative and absolute RBC parameters were compared between HAA and SLA. Absolute parameters were calculated by multiplying relative values with formula‐based estimated blood volume (BV‐e). Additionally, in two individual athletes, one HAA and one SLA, absolute parameters were also calculated with blood volume (BV) obtained by measurement with a dilution method (BV‐m). In men, HAA had a significantly higher hemoglobin (Hb) concentration (+7.8%; *p* = 0.001) and total Hb mass per kg body weight (BW) (+12.0%; *p* = 0.002). When not corrected for BW, HAA had a lower, non‐significant, total Hb mass (−7.8%; *p* = 0.055). In women, no significant differences between HLA and SLA were observed. The two individual athletes showed that, based on BV‐m, in the HAA, total Hb mass and total Hb mass per kg BW were respectively 14.1% and 31.0% higher than in the SLA, whereas based on BV‐e, in the HAA, total Hb mass was 20.8% lower and total Hb mass per kg BW was only 2.4% higher. Similar inconsistencies were observed for total RBC count. Thus, different ways of parameter expression, and different methods of BV assessment for the calculation of absolute parameter values, influence the interpretation of blood tests in athletes, which may lead to misinterpretation and incorrect conclusions.

## 1 INTRODUCTION

In the last decades, East African athletes, who are born and raised at high altitude, have dominated in middle‐ and long‐distance running (Knechtle et al., 2016). One explanation for their success is related to favorable hypoxia‐induced hematological adaptations, among which an increase in red blood cell (RBC) mass, and consequently an increase in oxygen transport capacity (Sinex & Chapman, 2015). Athletes born and raised at sea level have also started training at (simulated) altitude to benefit from these adaptations (Bonetti & Hopkins, 2009; Sinex & Chapman, 2015).

RBC parameters are commonly used to study hematological differences between high‐altitude athletes (HAA) and sea‐level athletes (SLA) in order to explain the success of HAA, and also to monitor the effect of high‐altitude training in athletes. Researchers, coaches and clinicians often rely on standard routine hematological measurements. These are affordable, easy to collect, and results are quickly available. In this way, RBC parameters are usually expressed as relative parameters (concentration) rather than absolute parameters (total amount). However, relative parameters are affected by changes in blood volume (BV), and could therefore lead to misinterpretation and incorrect conclusions. Absolute parameters are not affected by changes in BV, and might therefore be preferred to compare HAA with SLA and to evaluate the effect of (simulated) altitude training.

In this unique case series of elite HAA and SLA, we describe how different ways of parameter expression, and different methods of BV assessment for the calculation of absolute parameter values, can affect the interpretation of blood tests. Furthermore, we propose recommendations for the best way of RBC parameter expression and BV assessment.

## 2 METHODS

### 2.1 Case series

In a group of 42 elite athletes (29 HAA and 13 SLA), RBC measurements were performed at sea level as part of standardized routine sports medicine examinations. They were all runners who ranked top eight in major international track and field events, such as world championships, Olympic Games or comparable elite level events. Athletes were considered HAA if they were born and raised at altitudes of 6500 feet (~2000 m) to 10.000 feet (~3000 m) above sea level. These athletes still lived at this altitude most of the time, and they only visited lower altitudes for competition. Athletes were considered SLA if they were born and raised below 650 feet (~200 m). Athletes were middle‐distance (800–3000 m) and long‐distance (from >3000 m to 42 km) runners, indicating mainly use of their aerobic or their combined aerobic‐anaerobic energy system during exercise. They were in a competition period or in a training period that they subjectively rated as moderate to high intensity.

Red blood cell measurements were performed in the period 2002–2016. Only measurements from the first available examination were used for this study. Measurements in HAA were performed within 1 week after arrival at sea level; measurements from SLA within 3 months after training at high altitude were excluded. The measurements were fairly evenly distributed over the years and seasons in the study period.

We conducted additional measurements in two individual athletes: one HAA and one SLA. The HAA was a 23‐year‐old male middle‐distance runner, born and living in Kenya at an altitude of 8000 feet (~2400 m), who performed at international top ten level. Measurements were performed 4 days after arrival from Kenyan altitude, where he had stayed in the previous 3 months. The SLA was a 21‐year‐old male middle‐distance runner, born and living in the Netherlands at sea level, who performed at European top ten level. He had not performed training at high altitude within 3 months before the measurements.

All athletes were in good health. To our knowledge, none of the athletes used medication or banned substances with influence on the RBC values. They were frequently tested in‐ and out‐of‐competition according to international regulations of the World Anti‐Doping Agency.

This study was conducted according to the guidelines laid down in the Declaration of Helsinki and the Medical Ethical Committee of the University Medical Center Utrecht approved the study (protocol number 02/192; reference number: U‐02‐12948). Written informed consent was obtained from all the athletes prior to the analyses for this study.

### 2.2 RBC parameters

Overnight fasted blood samples taken in sitting position before 10.00 a.m. were used. The following RBC parameters were measured in all athletes: hematocrit (Ht), hemoglobin (Hb) concentration ([Hb]), and RBC concentration ([RBC]). Blood samples were analyzed in one certified hospital laboratory, using standard operating procedures and two types of equipment (Sysmex XE‐2100 and Advia‐120). Before use both apparatus were extensively evaluated according to CLSI protocols in order to ensure comparability.

To calculate absolute parameter values, BV was estimated using Nadler's formula (Nadler et al., 1962): BV (L) = 0.604 + 0.367 * height (m)^3^ + 0.0322 * weight (kg) for men and BV (L) = 0.183 + 0.356 * height (m)^3^ + 0.0331 * weight (kg) for women. The measured concentrations were then multiplied with the formula‐based estimated BV to obtain the absolute parameter values. Absolute parameters were reported as total amount and as total amount per kg body weight (BW).

In order to obtain more insight in the validity of our calculated absolute parameters, we also conducted BV measurements with a dilution method using radioactive chromium (^51^Cr), in two individual athletes: one male HAA and one male SLA. This method is considered as the gold standard for BV measurement (International Committee for Standardization in Haematology, 1980). A standard solution with a known amount of chromium‐51 labelled red blood cells in a known volume was injected intravenously. After 30 minutes, when complete mixing had taken place, a blood sample was taken. The radioactivity in the blood sample was then compared with the radioactivity in the standard solution, and in conjunction with the Ht value, the unknown BV was calculated. A detailed description of this method and associated calculations can be found elsewhere (International Committee for Standardization in Haematology, 1980). In these two athletes, absolute parameters were calculated by multiplying the measured concentrations (1) with estimated BV using Nadler's formula (BV‐e) and (2) with measured BV using the ^51^Cr method (BV‐m).

### 2.3 Statistical analysis

In the group of 42 elite athletes, data were first checked for normality using a Shapiro–Wilk test and visual inspection of Q–Q normality plots. Because some parameters were not normally distributed, and because of the unequal numbers of HAA and SLA and the relatively small sample size, all RBC parameters are presented as medians with 25th–75th percentiles and were compared between HAA and SLA using a Mann‐Whitney *U*‐test. Analyses were performed for men and women separately and considered significantly different when *p* < 0.05. Statistical analyses were performed with SPSS software (Version 23, IBM, Armonk, NY, USA).

For the two individual athletes, a descriptive comparison was used.

## 3 RESULTS

Results from the group of 42 athletes are presented in Table 1. In men, HAA had a significantly higher Ht (+10.4%; *p* = 0.003), [Hb] (+7.8%; *p* = 0.001) and [RBC] (+10.7%; *p* = 0.004), and a significantly higher total Hb mass per kg BW (+12.0%; *p* = 0.002) and total RBC count per kg BW (+11.9%; *p* = 0.006) compared to SLA. Without correcting for BW, HAA had a lower, although nonsignificant, total Hb mass (−7.8%; *p* = 0.055) and total RBC count (−4.6%; *p* = 0.169) compared to SLA. In women, no significant differences between HLA and SLA were observed. Note that absolute values in this group were calculated using BV‐e.

**TABLE 1:** Characteristics and parameter values in a series of high‐altitude athletes (n = 29) and a series of sea‐level athletes (n = 13)

	Men				Women			
	High‐altitude athletes (n = 19)	Sea‐level athletes (n = 7)	Difference HAA ‐ SLA	*p*‐value	High‐altitude athletes (n = 10)	Sea‐level athletes (n = 6)	Difference HAA ‐ SLA	*p*‐value
Characteristics								
Age, years	25 (22–29)	29 (22–30)	−4 (16.0)	0.306	25 (23–28)	22 (20–28)	+3 (12.0)	0.181
Height, m	1.71 (1.68–1.75)	1.84 (1.79–1.90)	−0.13 (7.6)	0.000*	1.66 (1.59–1.68)	1.74 (1.64–1.79)	−0.08 (4.8)	0.056
Weight, kg	58.0 (54.0–61.0)	72.0 (67.5–74.0)	−14.0 (24.1)	0.000*	50.0 (45.5–54.1)	54.5 (49.0–58.5)	−4.5 (9.0)	0.147
Parameters								
Expressed as concentration								
Ht, L/L	0.48 (0.46–0.49)	0.43 (0.42–0.46)	+0.05 (10.4)	0.003*	0.41 (0.39–0.41)	0.41 (0.39–0.43)	−	0.635
[Hb], g/dL^†^	16.6 (15.6–17.2)	15.3 (14.5–15.8)	+1.3 (7.8)	0.001*	13.8 (13.4–14.2)	13.6 (13.3–14.3)	+0.2 (1.4)	0.713
[RBC], 10¹²/L	5.6 (5.1–5.8)	5.0 (4.7–5.2)	+0.6 (10.7)	0.004*	4.7 (4.4–4.8)	4.4 (4.3–4.7)	+0.3 (6.4)	0.093
Expressed as total amount								
Total Hb mass, g^†^	715.3 (669.6–763.6)	770.8 (721.2–840.5)	−55.5 (7.8)	0.055	472.7 (456.3–484.6)	544.7 (454.9–580.0)	−72.0 (15.2)	0.147
Total RBC count, 10^12^	23.9 (22.9–25.3)	25.0 (23.8–27.4)	−1.1 (4.6)	0.169	16.1 (14.8–16.3)	17.3 (14.5–19.0)	−1.2 (7.4)	0.263
Expressed as total amount per kg BW								
Total Hb mass per kg BW, g^†^	12.5 (11.5–13.1)	11.0 (10.4–11.6)	+1.5 (12.0)	0.002*	9.5 (9.0–10.1)	9.7 (9.3–10.1)	−0.2 (2.1)	0.635
Total RBC count per kg BW, 10^12^	0.42 (0.37–0.44)	0.37 (0.34–0.38)	+0.05 (11.9)	0.006*	0.32 (0.31–0.34)	0.31 (0.30–0.33)	+0.01 (3.1)	0.428
Blood volume (BV‐e^‡^), L	4.34 (4.15–4.52)	5.20 (4.88–5.44)	−0.86 (19.8)	0.000*	3.43 (3.33–3.56)	3.85 (3.38–4.16)	−0.42 (12.2)	0.073
Blood volume (BV‐e^‡^) per kg BW, L	0.075 (0.073–0.077)	0.072 (0.072–0.074)	+0.003 (4.0)	0.063	0.070 (0.066–0.072)	0.071 (0.069–0.071)	−0.001 (1.4)	0.562

Values of characteristics and parameters are presented as median (25th–75th percentile), differences between HAA and SLA are presented as median difference with percentages in parentheses.

* Significant difference between HAA and SLA (*p*‐value Mann‐Whitney *U* test <0.05).

† Conversion factor g/dl into mmol/L: 0.6206; conversion factor g into mmol: 0.062.

‡ Estimated using Nadler's formula: BV (L) = 0.604 + 0.367 * height (m)^3^ + 0.0322 * weight (kg) for men and BV (L) = 0.183 + 0.356 * height (m)^3^ + 0.0331 * weight (kg) for women.

Results from the two individual athletes are presented in Table 2. The HAA had a slightly higher Ht (+5.8%), [Hb] (+2.9%), and [RBC] (+10.3%) than the SLA. In the HAA, BV‐m was much higher than BV‐e (6.29 L vs 4.32 L), whereas in the SLA, the difference between BV‐m and BV‐e was small (5.57 L vs 5.38 L). Total Hb mass was 14.1% higher in the HAA compared to the SLA when using BV‐m for its calculation, whereas using BV‐e, total Hb mass was 20.8% lower in the HAA. After correcting for BW, total Hb mass was 31.0% higher in the HAA than in the SLA using BV‐m, whereas using BV‐e, total Hb mass was only 2.4% higher in the HAA. Similar inconsistencies were observed for total RBC count.

**TABLE 2:** Characteristics and parameter values in one high‐altitude athlete and one sea‐level athlete in whom blood volume was measured

Characteristics/parameters	High‐altitude athlete	Sea‐level athlete	Difference HAA – SLA
Characteristics			
Age, years	23	21	+2 (8.7)
Height, m	1.71	1.88	−0.17 (9.9)
Weight, kg	58.4	72.5	−14.1 (24.1)
Parameters			
Expressed as concentration			
Ht, L/L	0.52	0.49	+0.03 (5.8)
[Hb], g/dL^†^	17.1	16.6	+0.5 (2.9)
[RBC], 10¹²/L	5.8	5.2	+0.6 (10.3)
Expressed as total amount			
(based on BV‐e^‡^)			
Total Hb mass, g^†^	738.7	892.6	−153.9 (20.8)
Total RBC count, 10¹²	25.1	28.0	−2.9 (11.6)
(based on BV‐m^§^)			
Total Hb mass, g^†^	1075.1	924.0	+151.1 (14.1)
Total RBC count, 10¹²	36.5	28.9	+7.6 (20.8)
Expressed as total amount per kg BW			
(based on BV‐e^‡^)			
Total Hb mass per kg BW, g^†^	12.6	12.3	+0.3 (2.4)
Total RBC count per kg BW, 10¹²	0.43	0.39	+0.04 (9.3)
(based on BV‐m^§^)			
Total Hb mass per kg BW, g^†^	18.4	12.7	+5.7 (31.0)
Total RBC count per kg BW, 10¹²	0.62	0.40	+0.22 (35.5)
Blood volume (BV‐e^‡^), L	4.32	5.38	−1.06 (24.5)
Blood volume (BV‐e^‡^) per kg BW, L	0.074	0.074	−
Blood volume (BV‐m^§^), L	6.29	5.57	+0.72 (11.4)
Blood volume (BV‐m^§^) per kg BW, L	0.108	0.076	+0.032 (29.6)

Differences between HAA and SLA are presented as absolute differences with percentages in parentheses.

† Conversion factor g/dl into mmol/L: 0.6206; conversion factor g into mmol: 0.062.

‡ Estimated using Nadler's formula: BV (L) = 0.604 + 0.367 * height (m)^3^ + 0.0322 * weight (kg) for men and BV (L) = 0.183 + 0.356 * height (m)^3^ + 0.0331 * weight (kg) for women.

§ Measured using the ^51^Cr method.

## 4 DISCUSSION

This unique case series shows marked differences in RBC parameters between elite HAA and SLA. But more importantly, our results also demonstrate that both the extent of these differences and the direction (higher or lower values) largely depend on how the parameter is expressed (relative or absolute, corrected for BW or not). Furthermore, absolute RBC parameters also depend on the method used for BV assessment (BV‐e or BV‐m) to calculate the absolute values.

### 4.1 HAA versus SLA

Only few articles have been published in which RBC parameters between HAA and SLA were compared within one study (Prommer et al., 2010; Saltin et al., 1995; Vergouwen et al., 1999). Some results of these studies are in agreement, whereas other results are contradictory to our observations. All these studies are difficult to compare because of differences in methodology and design. The large differences in methodology show that there is need for consensus on how to measure and express RBC parameters in athletes.

### 4.2 Effect of different use of parameter expression and BV assessment

In the group of male athletes, HAA had a significantly higher [Hb] and [RBC] compared to SLA, whereas results were in opposite direction when expressed as total amount (using BV‐e for the calculation of total amounts): total Hb mass and total RBC count were lower in HAA. When corrected for BW, total Hb mass per kg and total RBC count per kg were higher in HAA compared to SLA, and these differences were larger in comparison to expression as concentration.

For the two individual athletes, total Hb mass and total RBC count were higher in the HAA compared to the SLA when using BV‐m for its calculation, whereas the differences were in opposite direction when using BV‐e: total Hb mass and total RBC count where then lower in the HAA. After correcting for BW, the differences between HAA and SLA were in the same direction using either method, i.e., higher values for the HAA; however, the differences were much larger using BV‐m compared to using BV‐e.

An explanation for these observations in the two individual athletes is that in the HAA, BV‐m was much higher than BV‐e, whereas in the SLA, the difference between BV‐m and BV‐e was small. The large difference observed in the HAA between BV‐m (using the gold standard ^51^Cr method) and BV‐e, clearly shows that using Nadler's formula is less accurate. Another disadvantage of formula‐based estimation is that it does not take into account changes in BV. Moreover, studies have shown that people native to high altitude have a larger BV per kg BW compared to people from sea level (Sanchez et al., 1970), which was also observed in the current study when using BV‐m. It is likely that, using BV‐e to calculated absolute parameters, parameters values are underestimated in HAA.

### 4.3 Strengths and limitations

Although the sample size is small, this study describes the results of a unique study population of world class athletes. The invasiveness of the ^51^Cr method is the reason that we collected data of measured BV for only one HAA and one SLA, and results should therefore be interpreted with caution. We included measurements that took place under similar conditions of athletes, for example, regarding running distance, competition level, and training period; however, we cannot rule out some variation in these conditions, nor can we exclude some seasonal variation or some effects of travelling.

### 4.4 Recommendations

We recommend not to use relative RBC parameters to investigate hematological differences between HAA and SLA, but to use absolute RBC parateres, and to express the absolute values per kg BW.

In order to calculate absolute values per kg BW, we recommend to use measured BV rather than formula‐based estimated BV. Although the ^51^Cr method used in this study is considered as the gold standard for BV measurement (International Committee for Standardization in Haematology, 1980), the optimized CO‐rebreathing method is nowadays the default and least burdensome method, with measurement errors comparable to the ^51^Cr method (Gore et al., 2005; Schmidt & Prommer, 2005).

These recommendations may also be applicable for monitoring the effect of high‐altitude training in athletes. However, the optimized CO‐rebreathing method might still not always be available in sports (medicine) practice. Future effort should be targeted at developing an easy and accurate BV measurement method with quickly available results. Since this is not yet available, it remains impossible to recommend a feasible and reliable method to measure the effects of high‐altitude training in blood.

In conclusion, different ways of parameter expression, and different methods of BV assessment for the calculation of absolute parameter values, influence the interpretation of blood tests in athletes. Inappropriate parameter expression or BV assessment may lead to misinterpretation and incorrect conclusions.

## CONFLICT OF INTEREST

The authors declare no conflict of interest.

## AUTHOR CONTRIBUTIONS

All authors have given substantial contributions to the design of the manuscript. P.C.J.V. provided the data. A.M.B. analyzed the data, all authors interpreted the results. A.M.B. wrote the manuscript, all authors critically reviewed it. All authors have read and approved the final version of the manuscript.
